# Assessment of narrow-diameter implant survival for single-crown rehabilitation in the molar area

**DOI:** 10.6026/973206300222201

**Published:** 2026-04-30

**Authors:** Alisha Singh, Priyadershini A Rangari, Ranjeet Kumar Chaudhary, Neelam Singh, Kamal Nayan, Udaybhan Singh

**Affiliations:** 1 Department of Dentistry, ESIC Medical College and Hospital, Indore, Madhya Pradesh, India; 2 Department of Dentistry, Nandkumar Singh Chouhan Government Medical College, Khandwa, Madhya Pradesh, India; 3 Department of Dentistry, Shri Krishna Medical College Hospital, Muzaffarpur, Bihar, India; 4 Department of Prosthodontics and Crown and Bridge, IDST Dental College, Kadrabad, Modinagar, Ghaziabad, Uttar Pradesh, India; 5 Department of Oral & Maxillofacial Surgery, R. R. Dental College & Hospital, Udaipur, Rajasthan, India

**Keywords:** Molar region, narrow alveolar ridges, narrow diameter implants (NDI), standard diameter implants (SDI)

## Abstract

Literature lacks substantial data on survival rates of narrow-diameter dental implants (<3.75 mm) supporting single crowns in posterior molar regions under high masticatory loads. This randomized study compared clinical outcomes in 80 adults receiving narrow- diameter (n=40) versus standard-diameter (≥3.75 mm, n=40) implants for single molar crowns, assessing BOP, PD, mobility and crestal bone levels at 6 and 12 months. Narrow implants showed significantly less crestal bone loss at 6 months (p=0.001) and 12 months (p<0.001) than standard implants; BOP was lower in the narrow group at 3/6 months (p=0.01), though not significant at study end (p=0.42). PD increase was higher in narrow implants at 6/12 months (p<0.001), but implant mobility remained comparable between groups. Thus, we show by confirming equivalent 1-year success of narrow-diameter implants in molars, expanding minimally invasive options for compromised ridges despite limited prior evidence.

## Background:

Recently, dental implants have become one of the most widely accepted methods for replacing missing teeth, owing to their various advantages. These advantages include improved mastication ability and efficacy, better esthetics and acceptance by subjects undergoing it and preservation of alveolar bone levels. In the load-bearing posterior regions of the jaw, such as molars and premolars, SDI or standard diameter implants are usually preferred by clinicians who have a diameter of ≥3.75mm as they reduce the stress in the crestal areas, improve hygiene, increase implant to bone contact and decrease the associated risk for implant fracture [1]. There are certain clinical situations during dental implant placement in which the ridges where the implant is to be placed are not adequate in dimension for the placement of standard-diameter implants. It can be corrected to an extent using horizontal ridge augmentation to widen narrow alveolar ridges; however, it requires extended time, additional cost and is associated with various complications. As a result, many subjects are in denial about the invasive nature of the procedure. Another technique used for widening the edentulous ridge is ridge splitting. This has low predictability for the mandibular arch, particularly in the posterior areas, owing to a high likelihood of osseous plate fracture and decreased elasticity. These factors prompt clinicians to accept and widely use narrow-diameter dental implants [2]. Narrow-diameter implants have a diameter of ≤3.5 mm. These were initially used in the anterior region and depicted high success rates in areas with less occlusal load. The high success rate in the anterior area, improved implant surface and use of strong titanium alloys have led to their extension into the posterior areas of the jaws. The use of narrow- diameter implants has also been advocated in splined prostheses and overdentures. Recent studies in the literature assessing narrow- diameter implants to support a single crown in posterior jaw regions have depicted good and acceptable clinical outcomes [3]. Various studies in the literature have reported comparable results with narrow- and standard- diameter implants placed to support splinted and single crowns, with the majority of implants placed in the molar region. Also, marginal bone-level changes and soft- and hard-tissue peri-implant conditions around standard- and narrow-diameter implants are reported to be comparable for supporting a single crown. The available literature provides scarce evidence on narrow-diameter dental implants placed in the posterior region to support a single crown [4]. Therefore, it is of interest to investigate the survival of narrow-diameter implants supporting single-crown rehabilitation in the molar area.

## Materials and Methods:

This prospective clinical comparative study investigated the survival of narrow-diameter implants supporting single-crown rehabilitation in the molar area. The study considered subjects with missing single molar teeth and opted for dental implant replacement at the Department of Periodontology of the Institute during the study period. The subjects considered for the study were aged at least 20 years, had alveolar bone with dimensions sufficient for placing dental implants of diameter ≤3.5 mm, had narrow bone, consented to the study protocol and required an implant-supported crown in the molar areas. The subjects not considered were smokers, pregnant females, immunocompromised subjects, those using drugs that influence bone metabolism, those with a history of GBR (guided bone regeneration) at the planned site of implants and those with a very thin ridge. Subjects that were fit for the consideration criteria and a radiographic assessment was done for the determination of pathology and the height of the bone. Scaling was done, followed by home care instructions for oral hygiene. The edentulous area was thoroughly assessed, followed by palpation to assess any deformity, determination of the location and depth of lingual concavity, if any and morphological assessment. Ridges presenting with buccal concavity, suspected of dehiscence, were not considered. Intraoral radiographic and clinical evaluation were sufficient for assessing the edentulous area and hence CBCT (cone-beam computed tomography) was not compulsory [5]. Local anesthesia was considered for the surgery, followed by reflection of a mucoperiosteal flap to expose the crestal bone and insert narrow-diameter implants at the equicrestal level in the mandibular molar region, according to the manufacturer's standard drilling protocol. This was followed by repositioning the flap and suturing without tension in primary closure. Analgesics and antibiotics were prescribed. Two study groups were formed to divide the subjects into a control group (n=40) that received standard-diameter implants (3.75-4.2mm) and a test group (n=40) that received narrow- diameter implants (≤3.5mm). The parameters considered at the time of prosthesis insertion (baseline), 6 months and 12 months, including CBL (crestal bone levels), were assessed in mm from intraoral periapical radiographs. Reference points considered were the first bone to implant contact and the implant shoulder. Adequate magnification and light source were used for assessing the radiographs [6, 7]. Means for the distal and mesial values were computed to obtain one value per implant. Bone loss was taken as a negative change for the distance from the baseline value to further follow-up. PD (probing depth) was recorded at four sites for every implant using a Hu Friedy graduated periodontal plastic probe [8]. Mechanical testing was used to assess the implant mobility in the horizontal direction. Additionally, tapping was performed on the occlusal implant surface, where no mobility was considered to indicate solid sound and was graded as the presence or absence of mobility. Implant stability after placement was associated with implant survival [9]. Data were analyzed statistically using both parametric and nonparametric methods. The Wilcoxon rank test was used for intra-group comparisons at various time intervals and the Mann- Whitney U test for intergroup comparisons on the study data. The chi-square test was used to evaluate implant mobility. Considering a 95% confidence interval, statistical significance was assessed at a p-value <0.05.

## Results:

Results from the present study showed that mean crestal bone loss at baseline was 0.43±0.24, increasing to 0.72±0.21 and 0.93±0.24 at 6 and 12 months, respectively and was significant in the control group (p < 0.001). Mean CBL was 0.37± 0.15 at baseline in subjects from the test group, increasing significantly to 0.16±0.13 and 0.46±0.25 at 12 months, respectively, with p-values of 0.001 and <0.001, respectively. Intergroup assessment was non-significant between the two groups at 12 months, with p=0.11 (Tables 1 and 2 - see PDF). It was noted that in the control group, mean probing depth was 0.91±0.06 at baseline, increasing significantly to 1.15±0.25 and 1.36±0.32mm at 6 and 12 months, respectively. The mean difference from baseline at 6 months was 0.22±0.22 and at 12 months, 0.42±0.34, indicating statistical significance (p=0.001). Regarding the test group, mean probing depth at baseline was 0.95±0.05mm, increasing to 1.37±0.26 mm at 6 months and further to 1.58 ±0.41mm at 12 months. The mean differences at 6 and 12 months were 0.44±0.24 and 0.61±0.44 mm, respectively and were statistically significant (p-value <0.001). Intergroup comparison did not reveal any statistical significance with a p-value of 0.07 (Tables 3 and 4 - see PDF). For bleeding on probing, in the control group, a statistically non-significant difference was seen at 6 months and a significant difference at 12 months from baseline with 0.01±0.74 and 0.11±0.18, respectively and respective p-values of 0.13 and 0.01. Considering the test group, the mean difference from baseline to 6 months was non-significant, with a mean difference of 0.01 and a p-value of 0.33. From baseline to 12 months, a difference of 0.18 was observed, which was statistically significant (p=0.005). Intergroup comparison revealed a non-significant difference at 12 months with p=0.42 (Tables 5 and 6 - see PDF). At the end of the study, all the implants placed showed no mobility in either group (Table 7 - see PDF).

## Discussion:

The results of this study reported peri-implant soft and hard tissue parameters and responses that were comparable between narrow- diameter and standard-diameter implants. No implant was reported and mobility in both groups remained stable, indicating a good survival rate. A vital criterion for assessing the success of dental implants is peri-implant marginal bone integrity. Bone loss around dental implants initiates at the time of seal disruption between the cover screw and the implant and usually stabilizes post-loading after 6 months. Hence, implant success is now governed by 0.5mm bone loss around the implant after 6 months of loading. Implants that have > 0.5mm bone loss after 6 months of loading are at higher risk of future bone loss, as suggested by Galindo Moreno *et al.* in (2022) [10]. In this study, mean bone loss at 6 months in the test and control groups was 0.16 ±0.13 and 0.27±0.15, respectively, increasing to 0.46±0.25 and 0.53±0.19, respectively, at 12 months, showing an intergroup difference that was significant at 6 months (p=0.11) and non-significant at 12 months (p=0.01). These results were consistent with the findings of de Souza AB *et al.* in (2018), in which the authors also assessed peri-implant bone loss (PIBL) around standard- and narrow-diameter dental implants. After 3 years, PIBL showed no statistically significant difference in cumulative success and survival rates between the two groups, with a p-value of 0.576 [11]. Another study by Alrabiah *et al.* reported a non-significant difference in bone loss between standard- and narrow-diameter dental implants that replaced a single crown in the posterior region of the jaw at a 53-month recall [12]. Another study, in contrast, has reported greater bone loss around narrow-diameter implants in the molar region than in the premolar region, with statistical significance (p=0.01) [13]. However, no reason has been attributed in the study. In this study, bone remodelling was seen in both groups from baseline to 12 months. Bleeding on probing and probing depth are reliable parameters for assessing the health of peri-implant tissues; however, few studies report high levels of soft-tissue inflammation around narrow-diameter implants, which can be attributed to the large discrepancy between the implant diameter and the implant crown size, resulting in difficult hygiene and over-contoured restorations [14]. In the present research, all subjects maintained satisfactory oral hygiene. Various authors have reported healthy peri-implant tissues around narrow-diameter implants, including Zinsli *et al.* [15], Al-Shibani *et al.* [16], de Souza *et al.* [11] and Alrabiah *et al.* [12], where authors reported manageable oral hygiene and only minimal infection rates after narrow-diameter dental implant placement, as seen in the present study. No fracture was reported in any implant used in the study and no mobility was observed despite these implants' high susceptibility to fracture due to their narrow diameter. In a previous study by Zinsli *et al.* [15], two fractures were observed in narrow-diameter implants: one in an 8mm-long implant replacing a premolar with a wide occlusal table that fractured after 2 years of loading and another in a bruxism subject. The primary reason attributed to the fracture of narrow-diameter dental implants is overloading. The strength of narrow-diameter implants has greatly improved with the introduction of titanium alloys; in the present study, grade 5 titanium was used, which had a resorbable blast media surface. The success in narrow diameter dental implants is attributed to good quality of the bone, implant-protected occlusion (reduced cuspal inclination, eccentric contacts elimination and narrow occlusal table) and implant surface and design (rough surface implants, threaded, screw shape). Biomechanical factors have a more vital role than implant diameter for dental implant success, as described in the study by Gupta *et al.* (2025) [17], where the authors assessed complete edentulous rehabilitation, short span fixed partial dentures and single tooth replacement by implants and high failure was seen in short span fixed partial dentures. The results were comparable to those of the present study in standard and narrow-diameter dental implants.

## Conclusion:

Data shows that narrow-diameter implants that support a single crown placed in areas under high masticatory load achieve success and function comparable to those of standard-diameter implants. This is also true in subjects with inadequate bone dimensions to place standard- diameter dental implants. Candidates not suitable for invasive procedures, such as bone augmentation, narrow-diameter dental implants represent a valid alternative management.
